# Supplementary material for: Influence of Diabetes Mellitus on Postoperative Complications After Total Knee Arthroplasty: A Systematic Review and Meta-Analysis
Source: Medicina (Kaunas). 2024 Oct 26;60(11):1757. doi: 10.3390/medicina60111757 (PMC11595993; doi:10.3390/medicina60111757)
Supplement: Supplementary file 1 [file medicina-60-01757-s001.zip › Supplementary Table-S2.pdf]

Author(s):
Question: DM compared to Non-DM for TKA complication
Setting:
Bibliography:

Certainty assessment							№ of patients		Effect		Certainty	Importance
**№ of studies**	**Study design**	**Risk of bias**	**Inconsistency**	**Indirectness**	**Imprecision**	**Other considerations**	**DM**	**Non-DM**	**Relative (95% CI)**	**Absolute (95% CI)**		
**Periprosthetic joint infection**												
27	non-randomised studies	serious	not serious	not serious	not serious	none	-/599594	-/3154627	**RR 1.71** (1.46 to 2.00)	**0 fewer per 1,000** (from 0 fewer to 0 fewer)[a]	⊕◯◯◯ Very low	IMPORTANT
**Prosthesis revision**												
4	non-randomised studies	not serious	not serious	not serious	not serious	none	-/76197	-/567351	**RR 1.374** (1.237 to 1.526)	**0 fewer per 1,000** (from 0 fewer to 0 fewer)	⊕⊕◯◯ Low	IMPORTANT
**Cardiovascular disease**												
7	non-randomised studies	not serious	serious	not serious	not serious	none	-/473240	-/2429597	**RR 2.50** (1.50 to 4.17)	**0 fewer per 1,000** (from 0 fewer to 0 fewer)	⊕◯◯◯ Very low	IMPORTANT
**Cerebrovascular accident**												
4	non-randomised studies	not serious	not serious	not serious	not serious	none	2673/97192 (2.8%)	13644/770282 (1.8%)	**RR 2.38** (1.48 to 3.81)	**24 more per 1,000** (from 9 more to 50 more)	⊕⊕◯◯ Low	IMPORTANT
**Pneumonia**												
4	non-randomised studies	not serious	serious	not serious	not serious	none	-/465617	-/2035447	**RR 1.54** (1.15 to 2.07)	**0 fewer per 1,000** (from 0 fewer to 0 fewer)	⊕◯◯◯ Very low	IMPORTANT
**Urinary tract infection**												
4	non-randomised studies	not serious	serious	not serious	not serious	none	1878/97192 (1.9%)	11813/770282 (1.5%)	**RR 1.86** (1.07 to 3.26)	**13 more per 1,000** (from 1 more to 35 more)	⊕◯◯◯ Very low	IMPORTANT
**Sepsis**												
4	non-randomised studies	not serious	not serious	not serious	not serious	none	606/450144 (0.1%)	1648/1787332 (0.1%)	**RR 1.61** (1.46 to 1.78)	**1 more per 1,000** (from 0 fewer to 1 more)	⊕⊕◯◯ Low	IMPORTANT
**Deep vein thrombosis**												
10	non-randomised studies	serious	not serious	not serious	not serious	none	2425/770637 (0.3%)	8565/3207222 (0.3%)	**RR 1.58** (1.22 to 2.05)	**2 more per 1,000** (from 1 more to 3 more)	⊕◯◯◯ Very low	IMPORTANT
**Pulmonary embolism**												
4	non-randomised studies	not serious	not serious	not serious	serious	none	1534/475940 (0.3%)	5527/2012523 (0.3%)	**RR 1.13** (0.98 to 1.31)	**0 fewer per 1,000** (from 0 fewer to 1 more)	⊕◯◯◯ Very low	IMPORTANT
**In-hospital death or death within 3 months**												
10	non-randomised studies	not serious	not serious	not serious	not serious	none	-/684738	-/2678228	**RR 1.28** (1.02 to 1.60)	**0 fewer per 1,000** (from 0 fewer to 0 fewer)	⊕⊕◯◯ Low	IMPORTANT

CI: confidence interval; **RR:** risk ratio

Explanations

a. There is no full data of event number
