# Supplementary figures and images for: Influence of Diabetes Mellitus on Postoperative Complications After Total Knee Arthroplasty: A Systematic Review and Meta-Analysis
Source: Medicina (Kaunas). 2024 Oct 26;60(11):1757. doi: 10.3390/medicina60111757 (PMC11595993; doi:10.3390/medicina60111757)

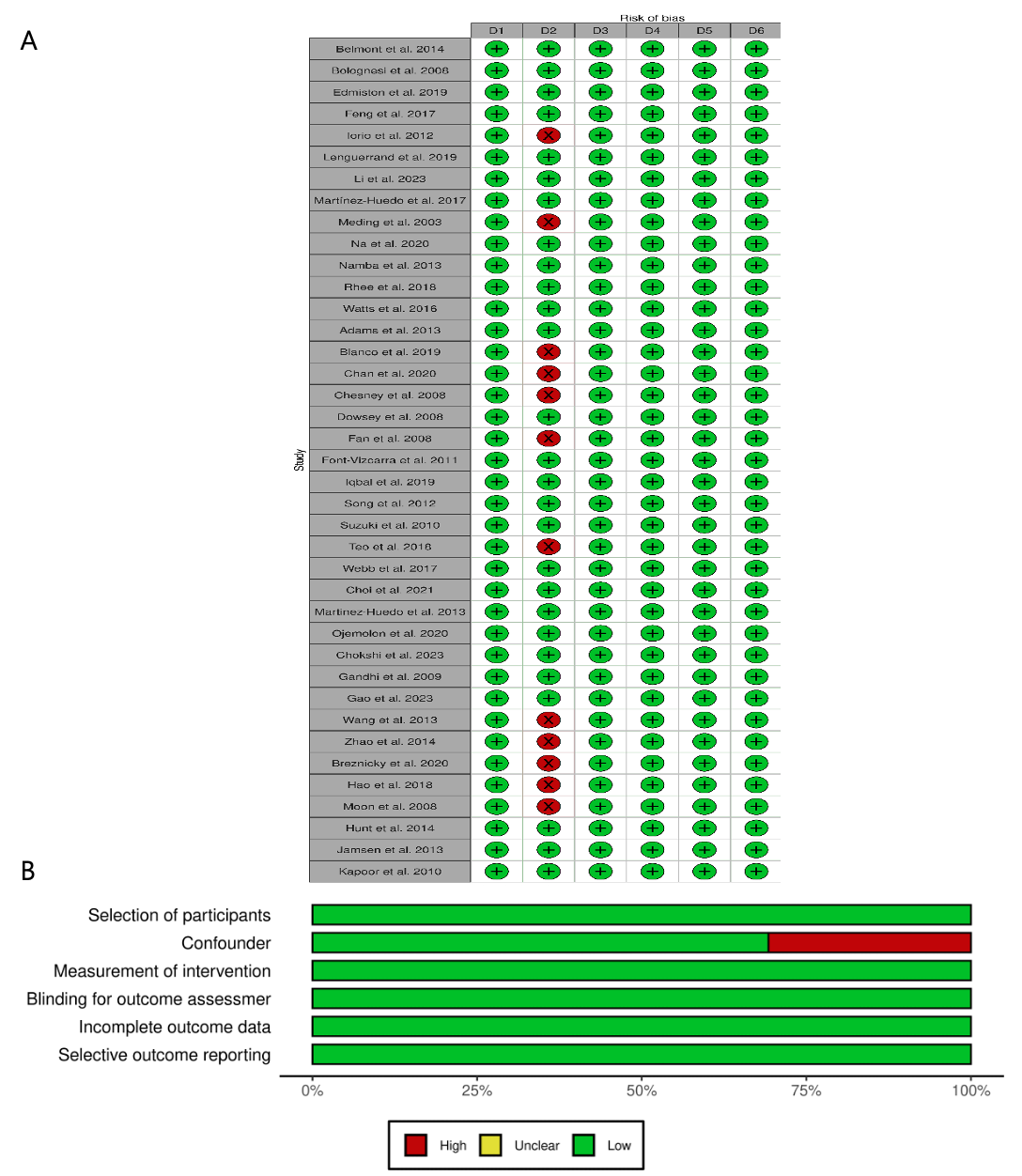

Supplement: Supplementary file 1 [file medicina-60-01757-s001.zip › Supplementary figure S1.png]

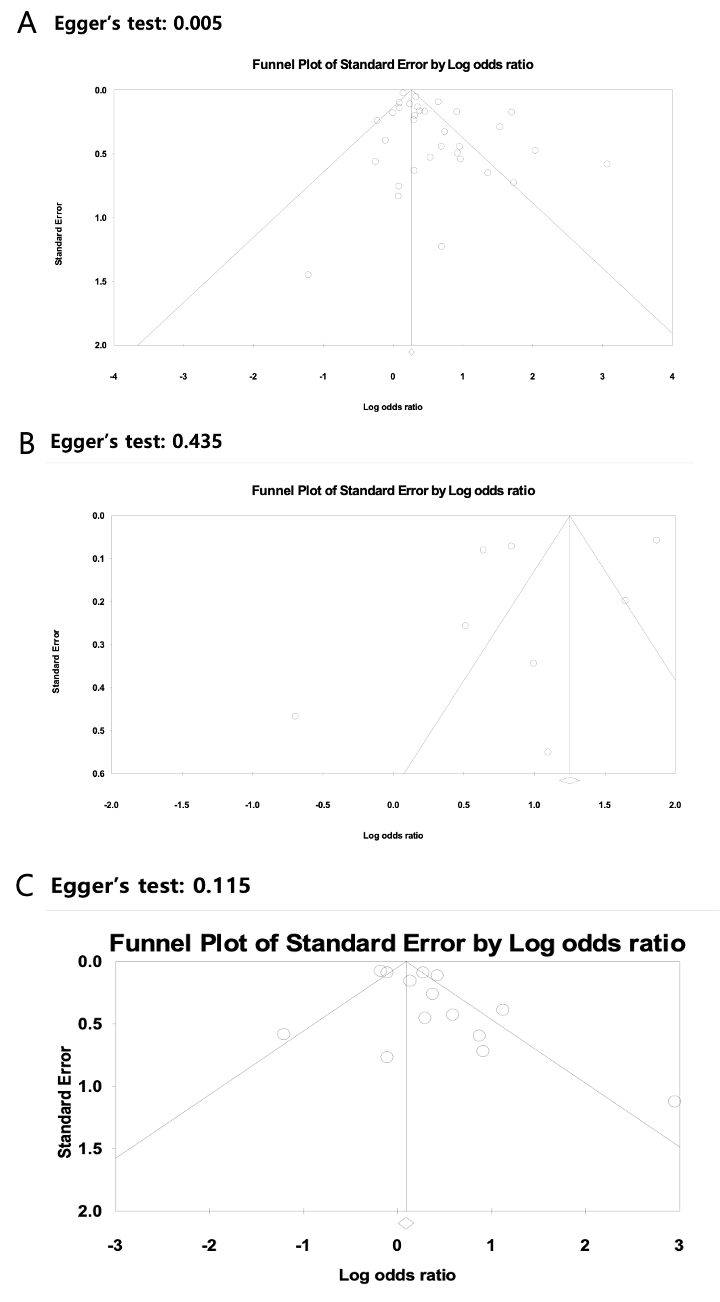

Supplement: Supplementary file 1 [file medicina-60-01757-s001.zip › Supplementary figure S2.png]

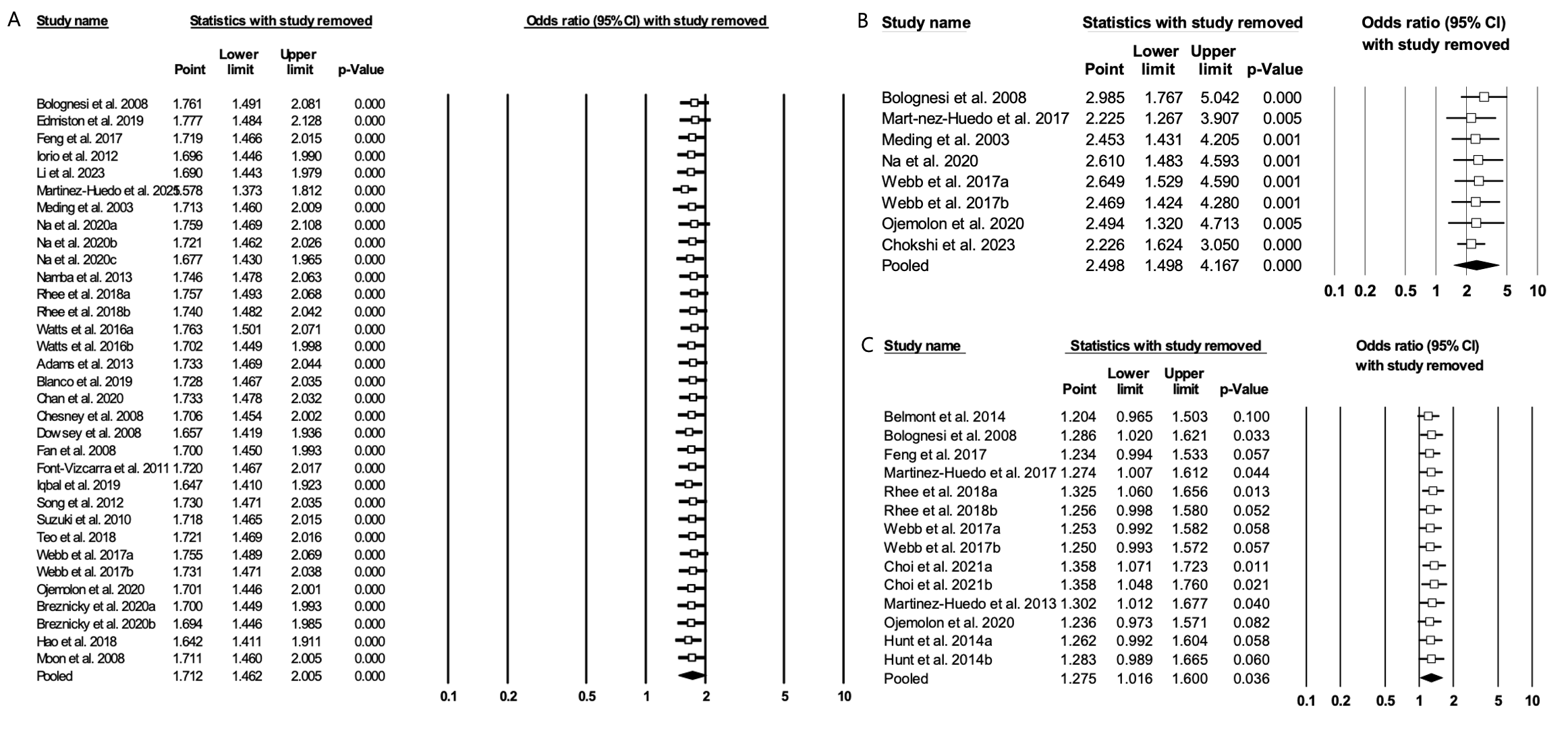

Supplement: Supplementary file 1 [file medicina-60-01757-s001.zip › Supplementary figure S3.png]
